# Single intrauterine demise in twin pregnancies: Analysis of 29 cases

**DOI:** 10.4274/tjod.35493

**Published:** 2015-12-15

**Authors:** Senem Yaman Tunç, Elif Ağaçayak, Neval Yaman Görük, Mehmet Sait İçen, Fatih Mehmet Fındık, Mehmet Sıddık Evsen, Abdulkadir Turgut, Serdar Başaranoğlu, Ahmet Yıldızbakan, Talip Gül

**Affiliations:** 1 Dicle University Faculty of Medicine, Department of Obstetrics and Gynecology, Diyarbakır, Turkey; 2 Memorial Hospitals, Clinic of Obstetrics and Gynecology, Diyarbakır, Turkey; 3 Fatih University Faculty of Medicine, Department of Obstetrics and Gynecology, İstanbul, Turkey

**Keywords:** Pregnancy, twin, single fetal death

## Abstract

**Objective:**

To evaluate the maternal and fetal demographic features and clinical aspects of twin pregnancies with single intrauterine demise.

**Materials and Methods:**

This retrospective study was conducted in Dicle University Faculty of Medicine, Department of Gynecology and Obstetrics between January 2008 and December 2013. There were a total of 594 twin deliveries in our hospital between the given dates. Twenty-nine of these cases were referred to our hospital by another health center because of a preliminary diagnosis of single intrauterine demise. Maternal age, parity, chorionicity, week of fetal death, gestational week at delivery, mode of delivery, birth weight, Activity, pulse, grimace, appearance, respiration scores, maternal fibrinogen levels at delivery and during pregnancy, stay in the neonatal intensive care unit, and obstetric complications were explored in these 29 cases of single intrauterine demise.

**Results:**

The mean age of the 29 patients who were provided antenatal follow-up and delivery services in our hospital was 29.9±6.5 years. Thirteen (44.8%) of the patients were monochorionic, whereas 16 (55.2%) were dichorionic. Intrauterine fetal death occurred in the first trimester in 6 pateints and in the second or third trimester in 23. In addition, 20 (69%) patients underwent cesarean section, whereas 9 (31%) had spontaneous vaginal delivery. Lastly, none of the patients had a maternal coagulation disorder.

**Conclusion:**

Twin pregnancies with single intrauterine death can lead to various complications for both the surviving fetus and the mother. Close maternal and fetal monitoring, and proper care and management can minimize complications.

## PRECIS:

Twin pregnancies with single intrauterine death can lead to various complications for both the surviving fetus and the mother. Hence, these pregnancies should be followed up in a tertiary center. Close maternal- and fetal monitoring, and proper care and management can minimize complications.

## INTRODUCTION

It has been reported that single intrauterine demise occurs in 5% of all twin pregnancies^(1)^. The etiology is unknown in the majority of cases; however, twin-to-twin transfusion syndrome, Rh incompatibility, chromosomal and congenital abnormalities, preeclampsia, umbilical vein thrombosis, single umbilical artery, abnormalities arising from the location of placenta, and umbilical cord and uterine malformations are the main causes of fetal death in the rest of the cases^(2,3,4)^. Single intrauterine demise may cause severe outcomes for the surviving fetus, especially in monochorionic twin pregnancies. Complications such as cerebral impairment, preterm labor and related sequelae, and subsequent death of the surviving fetus may occur in these cases^(5)^.

Ideal management is uncertain in twin pregnancies complicated by single intrauterine demise. The best time for delivery, frequency of antenatal tests, and maternal effects are still under debate^(6)^.

The purpose of this study was to explore the clinical features and fetal- and maternal outcomes of 29 twin pregnancies diagnosed as having single intrauterine demise.

## MATERIALS AND METHODS

This retrospective study was approved by Dicle University Faculty of Medicine, Board of Ethics (Ethics committee number: 356/10.06.2013). The study was conducted in Dicle University Faculty of Medicine, Department of Gynecology and Obstetrics between January 2008 and December 2013. Patients’ data on age, parity, chorionicity, week of fetal death, gestational week at delivery, mode of delivery, birth weight, Activity, pulse, grimace, appearance, respiration (APGAR) scores, maternal fibrinogen levels at delivery, stay in the neonatal intensive care unit (NICU), and obstetric complications were evaluated.

Twenty-nine twin pregnancies complicated by single intrauterine demise were included in this study. The presence of twin pregnancy and type of chorionicity were explored in the previous ultrasonography report of the first trimester. The patients were divided into two groups according to chorionicity. Group 1 consisted of dichorionic patients and group 2 consisted of monochorionic patients.

To assess fetal development and well-being, ultrasonography, Doppler ultrasonography, and Non-stress test (NST) were used in the follow-up period after the diagnosis was established. Monthly follow-ups were scheduled for patients with normal results. All patients with pregnancies of gestational age less than 34 weeks were given steroids for fetal lung maturity. Maternal fibrinogen and thrombocyte levels were assessed every 2-3 weeks during pregnancy and at the time of delivery. Maternal fibrinogen levels were measured using a clotting system (Sysmex CA 7000 System with SIEMENS reagents).

The diagnosis of Twin-to-twin transfusion syndrome (TTTS) was based on the visualization of a separating membrane, polyhydramnios-oligohydramnios sequence in the absence of other causes of abnormal amniotic fluid volume, size discordance, abdominal circumference, or weight discrepancy greater than 20%. Nonvisualization of donor’s bladder, abnormal fetal Doppler studies, hydrops or evidence of congestive heart failure were other recognized findings of TTTS.

Statistical package for Social Sciences version 15.0 (SPSS Inc., Chicago, IL, USA) was used for statistical analysis of data. Kolmogorov-smirnov test was used for the determination of data distribution. Data that were normally distributed were expressed as mean ± standard deviation, and non-normally distributed data were expressed as medians and ranges. The Mann-whitney U test and Chi-square test were used for comparisons between groups. A p value less than or equal 0.05 was considered statistically significant. Spearman’s correlation analysis was used to determine correlation coefficients.

## RESULTS

There were 594 twin deliveries occurred in our clinic between January 2008 and December 2013. Among these, 33 patients were diagnosed as having single intrauterine demise. Four of the 33 patients were excluded from the study on the grounds of inaccessible data. The remaining 29 twin pregnancies complicated by single intrauterine demise were included in the study. The mean age of the patients was 29.9±6.5 years. Ten (34.5%) of the patients were primiparous, whereas 19 (65.5%) were multiparous. Sixteen (55.2%) patients were dichorionic (group 1) and 13 (44.8%) were monochorionic (group 2).

Single intrauterine death occurred in the first trimester (before gestational week 14) in 6 patients and in the second or third trimester in 23 patients. Pregnancy was achieved spontaneously in 23 (79.3%) patients, by in-vitro fertilization (IVF) in 3 (10.3%), by intrauterine insemination (IUI) in one (3.4%), and through ovulation induction in 2 (6.9%) patients.

The mean gestational week at delivery was 33.7±3. The mean interval between fetal death and delivery was 77±54 days. The mean birth weight was 2116±719 grams. The mean 1-minute APGAR score was 5.8±1.5 and the mean 5-minute APGAR score was 8±1.2. The mean maternal fibrinogen level was 304.3±55.7 mg/dL. The maternal and fetal features of groups 1 and 2 are shown in Table 1.

In group 1, 3 patients had preeclampsia, 2 had preterm labor, and 2 had early membrane rupture. In addition, one patient had a previous diagnosis of antenatal gastroschisis. The infants of 3 pateints were taken to the NICU following delivery. In group 1, one newborn with gastroschisis was admitted to the intensive care unit. In addition, two newborns were admitted to the intensive care units because of prematurity. In group 2, one patient had preeclampsia, one had early membrane rupture, one had intrauterine growth restriction (IUGR) and severe oligohydramnios, and 2 patients had TTTS. The infants of the patients with IUGR and TTTS were taken to the NICU following delivery because of prematurity and IUGR. None of the 29 patients had perinatal mortality. Furthermore, no patients had a maternal coagulation disorder. No statistically significant difference was found between the groups in terms of maternal fibrinogen levels at delivery (p>0.05).

The frequency of preterm delivery was 46% among monochorionic twin pregnancies (group 2) and 43% among dichorionic twin pregnancies (group 1). The difference between the groups was not statistically significant (p>0.05).

There was a negative correlation between the mean gestational week of fetal death and the mean gestational week at delivery (Spearman’s rho=-0.380, p=0.042). Furthermore, no significant correlation was found between the mean gestational week of fetal death and mean fibrinogen levels (r=-0.317, p=0.107).

## DISCUSSION

Single intrauterine demise in twin pregnancies is a serious complication that occurs in 2.6-5% of all cases^(1,7)^. In a study conducted in Turkey, the incidence of this complication was reported to be 3.3%^(8)^. In the present study, the incidence was found to be 5.5%, which was slightly higher than the rates reported in the literature. The higher incidence might have been caused by the fact that our hospital is a tertiary healthy center that provides services for the whole region.

It is known that the risk of preterm delivery is increased in twin pregnancies complicated by single intrauterine demise. Hilmann et al.^(9)^ reported that the frequency of preterm delivery was 68% in monochorionic twin pregnancies and 54% in dichorionic twin pregnancies. Similarly, Ong et al.^(10)^ reported this frequency as 68% in monochorionic twin pregnancies and 57% in dichorionic twin pregnancies. The difference between the two groups was not found to be statistically significant. In agreement with the literature, in the present study the frequency of preterm delivery was 46% in monochorionic twin pregnancies and 43% in dichorionic twin pregnancies.

The mode of delivery should be selected according to the general obstetric condition of the mother in twin pregnancies complicated by single intrauterine demise. An early decision to perform a cesarean section owing to concerns of maternal coagulopathy should be avoided because it may cause prematurity-related problems for the fetus and increased morbidity for the mother^(11)^. In the present study, 69% (n=20) of the cases underwent cesarean section, whereas 31% (n=9) had spontaneous vaginal deliveries. Cesarean section was performed only in those patients with obstetric indications (10 patients with repeat cesarean sections, four patients with fetal distress, four patients with malpresentation, one patient with ablatio placenta, and one patient with fetal malformation).

Deveer et al.^(6)^ reported that the mean gestational age at diagnosis of a single fetal death was negatively correlated with gestational age at delivery. In our study, there was a negative correlation between the mean gestational week of fetal death and the mean gestational week at delivery; our findings were consistent with their study.

There are few cases in the literature with maternal disseminated intravascular coagulopathy that occurred after the intrauterine death of one fetus in multiple pregnancies^(12,13,14)^. Although some physicians administer short-term heparin to treat patients with hypofibronogenemia, it is known that it may resolve spontaneously without having to expose patients to treatment. In the present study, no patients had maternal coagulopathy. In addition, the difference between the two groups in fibrinogen levels at delivery and during pregnancy was not statistically significant.

Determination of chorionicity is important in twin pregnancies complicated by single intrauterine demise. Studies showed that prognosis was better in dichorionic twin pregnancies compared with monochorionic twin pregnancies^(7)^. Hilmann et al.^(9)^ reported that the frequency of mortality in the surviving fetus was 15% in monochorionic twin pregnancies and 3% in dichorionic twin pregnancies. Similarly, Ong et al.^(10)^ reported this frequency as 12% in monochorionic twin pregnancies and 4% in dichorionic twin pregnancies. In the present study, no intrauterine mortality occurred in the surviving fetuses.

According to the literature, the perinatal mortality of monochorionic twin pregnancies is double that of dichorionic twin pregnancies^(15)^. In our study, perinatal mortality was not observed in either group. This is likely to have been caused by the small number of patients in our study.

The optimum management of twin pregnancies complicated by single intrauterine demise is still uncertain. Many studies present insufficient data as to the management strategy in such cases. There are limited data in the literature as to the frequency of urgent deliveries following single intrauterine demise^(10)^. In our clinic, follow-up appointments take place every two weeks after gestational week 24 in twin pregnancies complicated by single intrauterine demise, and Doppler, non-stress test, and biophysical assessments are used to evaluate the current condition of the patient.

There are several limitations to our study. The relatively small number of patients and a lack of healthy twins as controls are ones to be noted. However, according to the literature, the loss of one twin in the first trimester does not appear to impair the development of the surviving twin(15). In our study, single intrauterine death in the first trimester (before gestational week 14) occurred in 6 patients. Another limitation of our study was the inclusion of these cases in the study.

In conclusion, twin pregnancies with single intrauterine death can lead to various complications for both the surviving fetus and the mother. Hence, these pregnancies should be followed up in a tertiary center. Close maternal- and fetal monitoring, and proper care and management can minimize complications. An early cesarean section should not be scheduled because of concerns of maternal coagulopathy, and prematurity should be avoided. Given the results of the present study, we recommend a conservative approach in twin pregnancies complicated by single intrauterine demise. However, larger studies are needed to determine the ideal management strategy in such patients.

## Figures and Tables

**Table 1:**
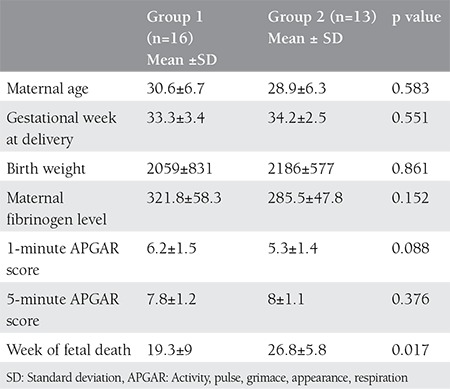
Maternal and fetal features of the groups
